# Out-Door Air and Exercise

**Published:** 1881-04

**Authors:** Felix L. Oswald


### OUT-DOOR AIR AND EXERCISE.

But the surest of all natural prophylactics is active exercise in the open air. Air is a part of our daily food and by far the most important part. A man can live on seven meals a week, and survive the warmest summer day with seven draughts of fresh water, but his supply of gaseous nourishment has to be renewed at least fourteen thousand times in the twenty-four hours. Every breath we draw is a draught of fresh oxygen, every emission of breath is an evacuation of gaseous recrements. The purity of our blood depends chiefly on the purity of the air we breathe, for in the laboratory of the lungs the atmospheric air is brought into contact at each respiration with the fluids of the venous and arterial systems, which absorb it and circulate it through the whole body; in other words, if a man breathes the vitiated atmosphere of a factory all day and of a close bedroom all night, his life-blood is tainted fourteen thousand times in the course of the twenty-four hours with foul vapors, dust and noxious exhalations. We need not wonder, then, that ill-ventilated dwellings aggravate the evils of so many diseases, nor that pure air should be almost a panacea.

Out-door life is both a remedy and a preventive of all known disorders of the respiratory organs; consumption, in all but the last stage of the *deliquium*, can be conquered by transferring the battle-ground from the sick-room to the wilderness of the next mountain-range. Asthma, catarrh, and tubercular phthisis, are unknown among the nomads of the intertropical deserts, as well as among the homeless hunters of our Northwestern Territories. Hunters and herders, who breathe the pure air of the South American pampas subsist for years on a diet that would endanger the life of a city dweller in a single month. It has been repeatedly observed that individuals who attained to an extreme old age were generally poor peasants whose avocations required daily labor in the open air, though their habits differed in almost every other respect; also that the average duration of life in various countries of the Old World depends not so much on climatic peculiarities or their respective degree of culture as on the chief occupation of the inhabitants; the starved Hindoo outlives the well-fed Parsee merchant, the unkempt Bulgarian enjoys an average longevity of forty-two years to the west Austrian citizen's thirty-five.—*From "Physical Education," by* DR. FELIX L. OSWALD, *in Popular Science Monthly for April.*

